# Microwave Breast Imaging Using Rotational Bistatic Impulse Radar for the Detection of Breast Cancer: Protocol for a Prospective Diagnostic Study

**DOI:** 10.2196/17524

**Published:** 2020-10-19

**Authors:** Shinsuke Sasada, Norio Masumoto, Hang Song, Akiko Emi, Takayuki Kadoya, Koji Arihiro, Takamaro Kikkawa, Morihito Okada

**Affiliations:** 1 Department of Surgical Oncology, Research Institute for Radiation Biology and Medicine Hiroshima University Hiroshima Japan; 2 Research Institute for Nanodevice and Bio Systems Hiroshima University Higashi-hiroshima Japan; 3 Department of Anatomical Pathology Hiroshima University Hospital Hiroshima Japan

**Keywords:** breast cancer, microwave imaging, diagnostic accuracy, screening, ultra-wideband radar

## Abstract

**Background:**

Mammography is the standard examination for breast cancer screening; however, it is associated with pain and exposure to ionizing radiation. Microwave breast imaging is a less invasive method for breast cancer surveillance. A bistatic impulse radar–based breast cancer detector has recently been developed.

**Objective:**

This study aims to present a protocol for evaluating the diagnostic accuracy of the novel microwave breast imaging device.

**Methods:**

This is a prospective diagnostic study. A total of 120 participants were recruited before treatment administration and divided into 2 cohorts: 100 patients diagnosed with breast cancer and 20 participants with benign breast tumors. The detector will be directly placed on each breast, while the participant is in supine position, without a coupling medium. Confocal images will be created based on the analyzed data, and the presence of breast tumors will be assessed. The primary endpoint will be the diagnostic accuracy, sensitivity, and specificity of the detector for breast cancer and benign tumors. The secondary endpoint will be the safety and detectability of each molecular subtype of breast cancer. For an exploratory endpoint, the influence of breast density and tumor size on tumor detection will be investigated.

**Results:**

Recruitment began in November 2018 and was completed by March 2020. We anticipate the preliminary results to be available by summer 2021.

**Conclusions:**

This study will provide insights on the diagnostic accuracy of microwave breast imaging using a rotational bistatic impulse radar. The collected data will improve the diagnostic algorithm of microwave imaging and lead to enhanced device performance.

**Trial Registration:**

Japan Registry of Clinical Trials jRCTs062180005; https://jrct.niph.go.jp/en-latest-detail/jRCTs062180005

**International Registered Report Identifier (IRRID):**

DERR1-10.2196/17524

## Introduction

### Background

The incidence of breast cancer is increasing, and the breast is the leading cancer location for women both worldwide and in Japan [1,2]. Mammography has been demonstrated to be effective in reducing breast cancer mortality and has become a standard screening method [3]. However, the screening rate of breast cancer varies greatly across countries; in Japan, it was 42.3% in 2016, which is lower than that in other developed countries [4,5]. The reasons for this lower rate of breast cancer screening could be the experience of pain by compression and exposure to ionizing radiation in mammography [6,7]. Therefore, painless and radiation exposure–free methodologies are required for the screening of breast cancer. In addition, mammography relies on the contrast in the X-ray attenuation coefficient between a normal mammary gland and malignant tissue, which can be as small as 1:1.1 [8]. This low contrast leads to lower sensitivity of mammography screening, particularly in dense breast tissue [6,7,9,10].

### Microwave Breast Imaging

Microwave breast imaging has developed significantly in the recent years [10,11]. The physical basis for microwave breast imaging consists of the difference in dielectric properties between the normal breast and cancerous tissues. The normal breast primarily consists of adipose tissue (low dielectric properties) with dispersed distributions of mammary glands (higher dielectric properties). Cancer tissue has greater dielectric properties at a frequency of 0.5GHz-50 GHz. A high contrast between healthy and cancerous tissues has been previously reported [12-17]. Among microwave breast imaging systems, the radar-based technology that uses time of flight of the impulse radio ultra-wideband (IR-UWB) signal is widely applied due to the simplicity and robustness of calculation [18-23]. This approach can calculate the amount of scattered electromagnetic energy at any spatial position to reconstruct an energy map of the object. The radar-based imaging method was developed in 1998, and it is referred to as confocal microwave imaging [18,19]. Synthetic focusing was performed under the assumption that the dielectric constant of breast was homogeneous. The electromagnetic signals scattered from the target location were calculated based on the signal path between the transmitter and receiver antennas and the average propagation speed of microwave radiation. This method reconstructs an energy map, which shows the qualitative position of the highest intensity of the confocal image of the scattered signals. In addition, the bistatic radar system, which allows different aspects of a target to be visualized, can deliver more information than the monostatic systems [24-29].

Some microwave breast imaging systems have already been evaluated in clinical settings. The largest cohort studies have been conducted with 86 and 223 patients using the Multistatic Array Processing for Radiowave Image Acquisition (MARIA) system [26,30]. Results indicated that the sensitivity of breast cancer detection was 74%-75%. Studies using other systems have also been conducted, and the results from 150 females with abnormal mammograms indicated a mean increase in the image contrast of 150%-200% between abnormal and normal breast tissues [31-33]. However, these conventional prototypes use vector network analyzers, resulting in heavy instrumentation and high cost. Although some groups have developed compact microwave imaging systems [27-29,34,35], these systems are yet to be applied in clinical settings.

### Preliminary Work

We developed a prototype handheld bistatic radar–based system using complementary metal–oxide–semiconductor (CMOS)-integrated circuits [36-41]. In a clinical pilot study, this system showed a 100% sensitivity in 5 patients with breast cancer [42]. Information on specificity and causes of false positives using this microwave imaging technology has not yet been clarified. Therefore, we aim to determine the diagnostic performance of our in-house bistatic radar–based detector with patients with breast cancer or those with benign breast tumors.

### Purpose

This study has 3 major goals. It aims to assess the sensitivity and specificity of the system in screening for breast cancer, assess the sensitivity and specificity of the system screening for benign tumors, and investigate the impact of tumor biology based on the measured results.

## Methods

### Study Design

**Figure 1:**
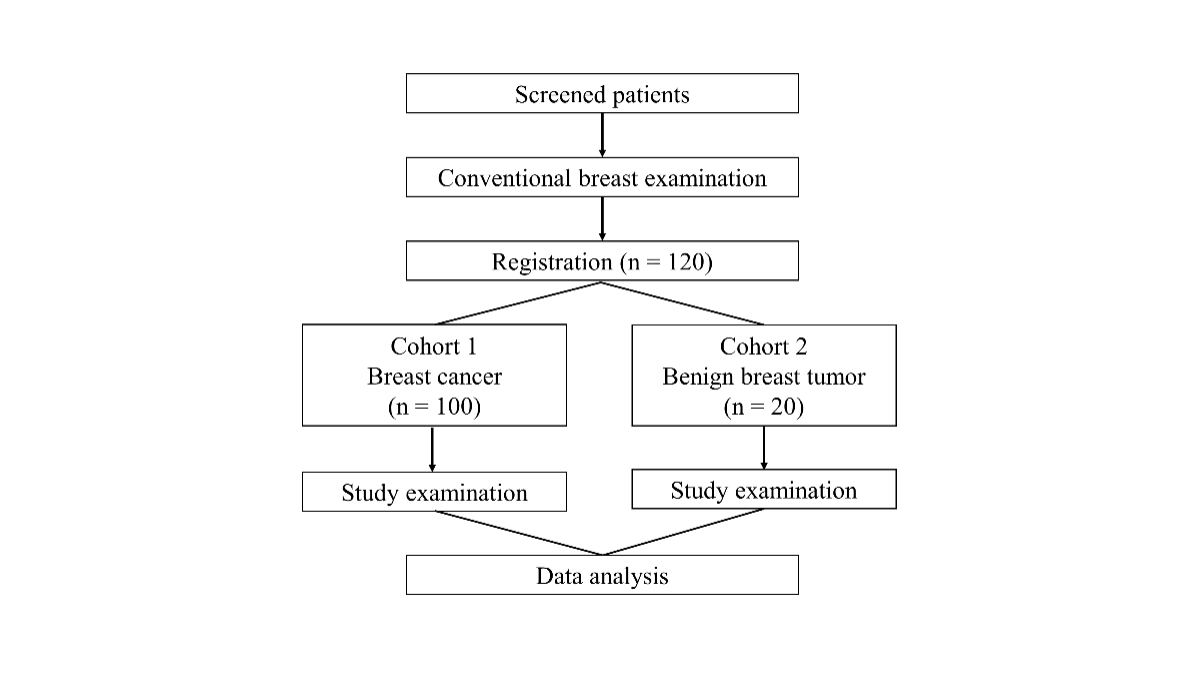
Study flowchart.

This study is a single-center, single-arm, prospective diagnostic study on women with breast tumors and will be performed as a specified clinical trial under the Japanese law on clinical research (the Clinical Trials Act). Women with breast cancer or benign breast tumors as diagnosed by conventional examinations were recruited for this purpose. An examination using the handheld bistatic radar–based breast imaging system will be performed before any treatment. The flowchart and time schedule for this study are shown in Figure 1 and Table 1, respectively.

**Table 1:** Time schedule of the study.

Procedure	Baseline	Enrollment (week 0)	Intervention and assessment (week 0 to week 8)
Clinical breast imaging^a^	✓		
Pathological examination	✓		
Informed consent		✓	
Confirmation of eligibility criteria		✓	
Study examination			✓
Safety			✓

^a^Conducted by mammography, breast ultrasonography, contrast-enhanced breast magnetic resonance imaging, or fluorodeoxyglucose positron emission tomography.

The Certified Review Board of the Hiroshima University Hospital approved this study (CRB6180006) on October 3, 2018 (version 1). All procedures will be in accordance with the Japanese law (the Clinical Trials Act), the Declaration of Helsinki, and comparable ethical standards. This study is covered by clinical research insurance, and compensation is provided. Written informed consents will be obtained from all study participants.

### Study Population

Participants were recruited from the Hiroshima University Hospital and were classified into 2 groups: cohort 1, comprising 100 women with histologically diagnosed breast cancer; and cohort 2, comprising 20 women with histologically or radiologically diagnosed benign breast tumors.

The inclusion criteria included (1) female patients older than 20 years; (2) those with histologically confirmed breast cancer (only for cohort 1); (3) those with confirmed breast tumor by 1 or more arbitrary imaging examinations, such as mammography, breast ultrasonography, contrast-enhanced breast magnetic resonance imaging, or fluorodeoxyglucose positron emission tomography; and (4) those who provide a written informed consent. The exclusion criteria included (1) history of chemotherapy or radiotherapy within 6 months prior to registration; (2) hypersensitivity to the acrylonitrile butadiene styrene (ABS) plastic resin; (3) presence of a pacemaker; or (4) a physician’s decision. Participant examination will be discontinued and their data will be removed in case of withdrawal of consent, ineligibility, inability to participate in the study due to disease progression or complications, death, induction of treatment before the study, or any other exclusion criterion deemed necessary by the investigators. Benign breast tumors will be diagnosed by the Breast Imaging Reporting and Data System (BI-RADS) (ultrasound category 2 or less), fine-needle aspiration, or core needle biopsy. The radiological images will be read by 2 experienced radiologists.

### Study Examination

**Figure 2:**
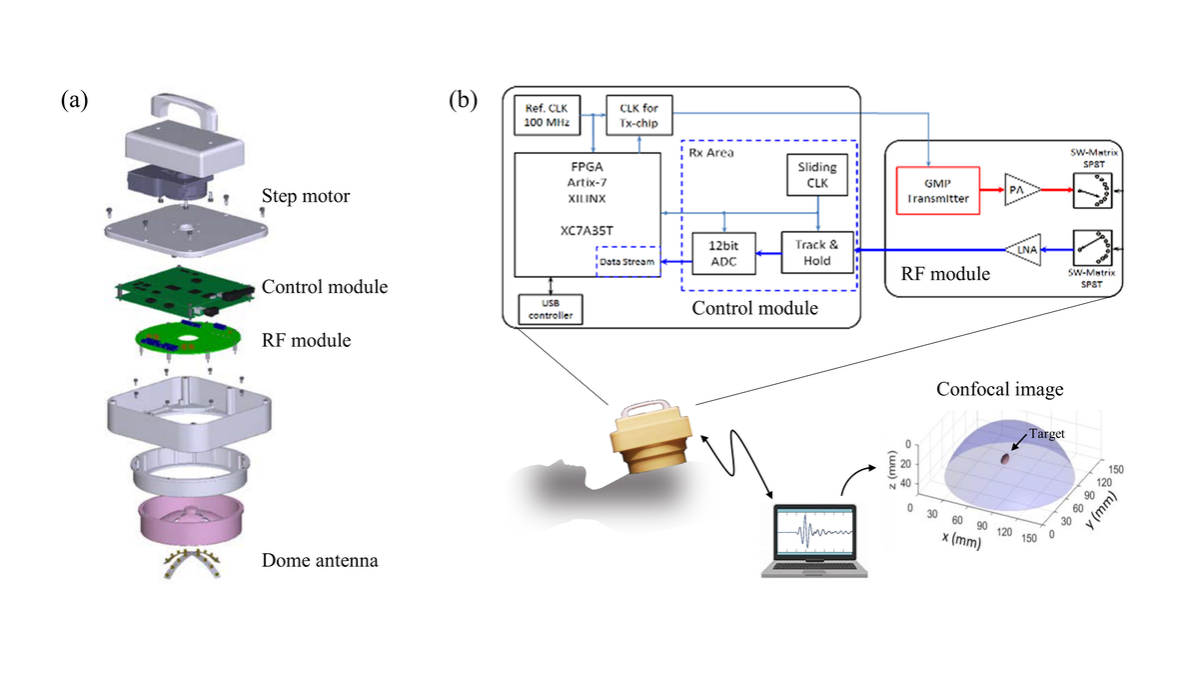
Structure of the microwave breast tumor detector. ADC: analog-to-digital converter; CLK: clock; FPGA: field-programmable gate array; GMP: Gaussian monocycle pulse; LNA: low noise amplifier; PA: power amplifier; Ref. CLK: reference clock; RF: radio frequency; RX: receiver; SP8T: single-pole 8-throw; SW: switching; TX: transmitter.

Measurements and weight of the handheld detector are 19.1 × 17.7 × 18.8 cm and 2 kg, respectively. It consists of a handle, step motor, control module, radio frequency module, and dome antenna array. The structure of the detector is depicted in Figure 2A. The system is composed of CMOS-integrated circuits that enable the generation and transmission of Gaussian monocycle pulse (GMP) trains and the control of the combination of a 4 × 4 cross-shaped dome antenna array, using a single-pole 8-throw switching matrix (SP8T-SW). The dome antenna array consists of 16 elements, and each antenna element is composed of a square slot set in a ground plane on 1 side of a Duroid RT 6010 substrate. The center frequency and the bandwidth of the ultra-wideband antenna are 6 GHz and 6.7 GHz, respectively. The dome shell is composed of ABS to hold the antennae in place. The dome antenna will be placed on each breast without any coupling liquid with the participant in a supine position and held by hand or supported by a stand to keep the device stationary. A transmitter antenna array emits GMP signals with the pulse duration of 160 picoseconds at a repetition frequency of 100 MHz to illuminate the breast tumor, and a receiver antenna receives the reflected signal. The antenna array rotates in 9 steps from 0° to 360° using a step motor. A single inspection can yield 40 sets of data with 2048 measurement points acquired within 5 minutes for each breast. The received signals are converted from analog to digital via a 12-bit analog-to-digital converter, and a confocal image is constructed (Figure 2B).

### Study Endpoints

The primary endpoint is the detection sensitivity and specificity for breast cancer or benign breast tumors by the screening system. Contralateral healthy breasts will also be measured in this study, and the diagnostic accuracy will be calculated on a per-breast basis. The secondary endpoints are sensitivity for breast cancer detection according to the different molecular subtypes, sensitivity for benign breast tumors according to histology, and feasibility of the examination. The biology of breast cancer will be pathologically evaluated by surgery or using collected biopsy specimens. For exploratory purposes, the influence of tumor size and breast density on the detectability of breast tumor will also be investigated.

### Sample Size Calculation

Sample size calculations were based on the sensitivity of breast cancer detection and the significance of breast cancer screening. The expected sensitivity was set at 80% with reference to the sensitivity of the Japanese mammography screening [43]. The required sample size was 171 breasts according to a width of the 95% CI being set to 0.12 at one-sided α=.05. Therefore, a sample size of 100 women (200 breasts) in cohort 1 was proposed.

### Data Security and Analysis

Data will be entered directly in a database and kept in a secure network computer in a locked office at the Hiroshima University. All information will be uniquely anonymized. The operational process of this study will be subject to annual monitoring, and no audit is required. The occurrence of serious adverse events will be reported to the Certified Review Board of the Hiroshima University Hospital or the Ministry of Health, Labor and Welfare of Japan. Raw data from measurements will be analyzed using customized software that was independently developed to control the operation of the detector. Statistical analysis will be performed using the R statistical software (R Foundation for Statistical Computing) environment.

## Results

Recruitment began in November 2018 and was completed by March 2020. We expect to submit the results for publication by summer 2021.

## Discussion

### Overview

Some microwave breast imaging systems have been previously developed and clinically investigated [11]. Of these, 2 systems were considered in clinical studies with large cohorts and suggested their potential utility in the clinical settings [30,32]. These studies focused solely on sensitivity, but the diagnostic accuracy (including specificity) of these systems has not been reported. To the best of our knowledge, this study is the first of its kind that focuses on both sensitivity and specificity of a microwave breast imaging system on a per-breast basis.

Our imaging system has several advantages in addition to the general features of microwave imaging. First, it is an easy-to-operate system, where an operator simply places the device in close contact with a breast and presses a start switch. No special training is required for the examinations, and the system is not affected by differences of technical skills of its operators. Second, this system places compact integrating antennae, excitation and acquisition hardware, and signal routing in a single unit. The high portability will provide a significant benefit for remote inspection [44]. Additionally, this compact system can be used repeatedly at low cost and is useful for monitoring applications. The advantages of this system make it suitable for breast cancer screening, although concerns regarding the production of mechanical heat and influence on pacemakers need to be assessed. In the prestage experiment, the temperature of the device after 30 minutes of operation was 30 degrees, and thus the burn risk was minimal. In addition, this study will exclude individuals with a pacemaker. Therefore, the risk associated with this study is low. Data obtained in this study will be helpful for future system improvements. This study will also provide important information on factors that lead to the false positives in breast lesions and differentiation between benign and malignant lesions.

### Limitations

This study targets breast tumor cases. The diagnostic accuracy of this screening system cannot be evaluated on a per-person basis in clinical settings. In addition, the data analysts will be blind to whether breast tumors are on the left or right because of the influence of the interpretation of the test data.

### Conclusions

We expect that this study will demonstrate the clinically meaningful diagnostic accuracy of the bistatic IR-UWB radar–based breast imaging system. We also hope that this study will propose a novel methodology for breast cancer screening and contribute to improvements in the current technical equipment.

## Abbreviations

ABS: acrylonitrile butadiene styrene
BI-RADS: Breast Imaging Reporting and Data System
CMOS: complementary metal–oxide–semiconductor
GMP: Gaussian monocycle pulse
IR-UWB: impulse radio ultra-wideband
MARIA: Multistatic Array Processing for Radiowave Image Acquisition
SP8T-SW: single-pole 8-throw switching matrix
